# Multiple mycobacterial antigens are targets of the adaptive immune response in pulmonary sarcoidosis

**DOI:** 10.1186/1465-9921-11-161

**Published:** 2010-11-23

**Authors:** Kyra A Oswald-Richter, Dia C Beachboard, Xiaoyan Zhan, Christa F Gaskill, Susamma Abraham, Cathy Jenkins, Daniel A Culver, Wonder Drake

**Affiliations:** 1 Department of Microbiology and Immunology, Vanderbilt University School of Medicine, Nashville, TN 37232-2363, USA; 2 Division of Infectious Diseases and Department of Medicine, Vanderbilt University Medical School, Nashville, TN 37232-2363, USA; 3 Respiratory Institute, Cleveland Clinic, Cleveland, OH 44195, USA; 4 Department of Biostatistics, Vanderbilt University Medical School, Nashville, TN 37232-2363, USA

## Abstract

**Introduction:**

Sarcoidosis is a multisystem granulomatous disease for which the association with mycobacteria continues to strengthen. It is hypothesized that a single, poorly degradable antigen is responsible for sarcoidosis pathogenesis. Several reports from independent groups support mycobacterial antigens having a role in sarcoidosis pathogenesis. To identify other microbial targets of the adaptive immune response, we tested the ability of CD4+ and CD8+ T cells to recognize multiple mycobacterial antigens.

**Methods:**

Fifty-four subjects were enrolled in this study: 31 sarcoidosis patients, nine non-tuberculosis mycobacterial (NTM) infection controls, and 14 PPD- controls. Using flow cytometry, we assessed for Th1 immune responses to ESAT-6, katG, Ag85A, sodA, and HSP.

**Results:**

Alveolar T-cells from twenty-two of the 31 sarcoidosis patients produced a CD4+ response to at least one of ESAT-6, katG, Ag85A, sodA, or HSP, compared to two of 14 PPD- controls (p = 0.0008) and five of nine NTM controls (p = 0.44), while eighteen of the 31 sarcoidosis subjects tested produced a CD8+ response to at least one of the mycobacterial antigens compared to two of 14 PPD- controls (p = 0.009) and three of nine NTM controls (0.26). Not only did the BAL-derived T cells respond to multiple virulence factors, but also to multiple, distinct epitopes within a given protein. The detection of proliferation upon stimulation with the mycobacterial virulence factors demonstrates that these responses are initiated by antigen specific recognition.

**Conclusions:**

Together these results reveal that antigen-specific CD4+ and CD8+ T cells responses to multiple mycobacterial epitopes are present within sites of active sarcoidosis involvement, and that these antigen-specific responses are present at the time of diagnosis.

## Introduction

Sarcoidosis is a granulomatous disease of unknown etiology, characterized by a Th1 immunophenotype. Immunological studies have shown that there are oligoclonal T cells in sarcoid bronchoalveolar lavage (BAL), suggesting that the response is antigen-specific [1,2]. Investigation into T cell receptors in BAL fluid has shown an AV2S3+ T cell expansion in the lung of sarcoidosis patients with HLA-DRB1*0301 and HLA-DRB3*0101 also supporting an antigen specific response in the sarcoid lung [3]. Multiple studies have shown an association between mycobacterial antigens and sarcoidosis. Song et al. found mycobacterial katG in sarcoidosis granulomas using both mass spectrometry and immunohistochemistry [4]. In addition, mycobacterial DNA has been found in sarcoid granulomas [5,6]. Studies have also shown T cell Interferon-γ (IFN-γ) producing responses to mycobacterial peptides in both peripheral blood mononuclear cells [7-12] and bronchoalveolar lavage fluid [9,13].

It has been suggested that sarcoidosis is a hypersensitivity reaction caused by prolonged exposure to a specific antigen [9]. However, we have shown in a previous study that in peripheral blood mononuclear cells, multiple antigens induce IFN-γ production [8], as well as that T cells from sarcoidosis BAL can produce an IFN-γ response to both mycobacterial ESAT-6 and KatG [14].

The purpose of this study is to assess if responses to multiple mycobacterial virulence factors are present in an active site of sarcoidosis involvement at the time of diagnosis. We investigated for immune response to actively secreted mycobacterial virulence factors that had previously been shown to elicit Th-1 immune responses [14]. We analyzed specific responses to mycobacterial early secreted antigenic target protein 6 (ESAT-6), catalase-peroxidase (katG), mycolyl transferase (Ag85A), superoxide dismutase A (sodA), and heat shock protein 70 (HSP) in BAL derived T cells.

## Materials and methods

### Study subjects

The study participants were recruited from The Cleveland Clinic (Cleveland, OH) and Vanderbilt University Medical Center (Nashville, TN). We prospectively enrolled patients who were undergoing bronchoscopy where sarcoidosis was a diagnostic consideration (Table 1). All subjects provided written informed consent that was approved by the appropriate Institutional Review Boards. Fifty-four patients were enrolled in this study: 31 sarcoidosis subjects, 14 disease controls with a negative purified protein derivative (PPD-) test, and 9 subjects with a nontuberculosis mycobacterial infection (NTM) such as *Mycobacterium avium *(Table 1, Additional File 1, Table S1). The PPD- disease controls were subjects with clinical syndromes consistent with sarcoidosis for which an alternative diagnosis was obtained such as Aspergillosis, fungal infection, lymphoma, lung cancer and pneumonia. None of these patients were diagnosed with fungal infection. Demographic characteristics of these patient groups are listed in Table 1 and Additional File1, Table S1. For the sarcoidosis subjects who underwent tuberculin skin testing at the discretion of their physician, all were skin test negative, and none have developed mycobacterial infection since the time of their bronchoscopy. Kruskal-Wallis analysis indicated there was a significant difference in age distributions across patient groups (p < 0.001; Table 1); a Pearson test performed across patient groups indicated no difference according to race or sex (Table 1; bi-racial subjects were omitted from the race analysis).

**Table 1:** Demographics of study population

Characteristics	Sarcoidosis	PPD-	NTM	p value
Number	31	14	9	NS
Sex, female/male	19/12	11/3	5/4	NS
Age (yr), median (min, max)	47 (25,64)	49 (24,61)	67 (51,75)	NS
Race	8AA:22C:1H/AA	3AA:10C:1A/S	1AA:8C	NS
Stage 0/I/II/III/IV	1/12/17/1/0	ND	ND	
IS at Bronch	12/19	ND	ND	
Lym (%), median (min, max)	15 (1, 59)	7 (1, 37)	14 (3, 66)	
Extrapulmonary disease, yes/no	7/24	ND	ND	
Smoking status	3AS:11EX:17NS	1AS:5EX:8NS	1AS:5EX:3NS	
CD4/CD8 ratio, median (min,max)	2.87 (0.56,70.54)	1.30 (0.14,36.57)	2.95 (0.49,9.1)	

Definition of abbreviations: NTM = non-Tuberculosis mycobacterial infection; NS = not significant, yr = years, min = minimum, max = maximum, ND = not done, A = Asian, AA = African American, C = Caucasian, H = Hispanic, S = Samoan; IS = immunosuppressant; Lym = lymphocyte; AS = active smoker; EX = ex-smoker; NS = never smoker. Statistical differences were assessed using the Wilcoxon rank sum test. The p values listed are for the comparison between sarcoidosis and PPD-. No significant difference was seen between sarcoidosis and NTM groups.

The subjects had their chest radiographs or chest computer tomography scans staged according to the method described by Scadding [15]: stage 0: no infiltrates or adenopathy,; stage 1: hilar adenopathy alone; stage 2: adenopathy plus infiltrates; stage 3: infiltrates alone; stage 4: fibrosis. Unlike the traditional Scadding system, these determinations were made by viewing the chest CT scan when one was available. Otherwise, the chest radiograph was used to perform this staging.

Histopathological diagnosis of sarcoidosis was confirmed by a pathologist (i.e. specimens from each patient had confluent non-caseating granulomas, with negative cultures and stains for bacteria, fungi and acid-fast bacilli), and no history of exposure to antigens known to cause granulomatous lung disease. Disease controls were subjects for whom an alternate diagnosis was obtained after bronchoscopy.

### BAL isolation and culture

BAL fluid was obtained from diagnostic bronchoscopy and centrifuged at 1500 rpm for 15 minutes. The BAL cell pellet was washed with RPMI 1640 supplemented medium. BAL cells were either stored in liquid nitrogen in freezing media for future analysis or analyzed immediately. The media used in all experiments was RPMI 1640 (Cellgro) supplemented with 10% FBS (Gemini Bio-Products), penicillin (50 U/ml; Cellgro), streptomycin (50 μg/ml; Cellgro), sodium pyruvate (1 mM; cellgro), and glutamine (2 mM; Cellgro). Freezing media consisted of 50% RPMI 1640 supplemented medium, 40% FBS, and 10% DMSO.

### Synthesis of Mycobacterium peptides

The two ESAT-6, two KatG, four Antigen 85A (Ag85A) peptides, and four SodA peptides were synthesized as described previously (Table 2) [16]. Each peptide was synthesized by solid-phase F-moc chemistry (Genemed Synthesis, San Diego, CA, USA), to a purity of > 70%. Identity was confirmed by mass spectroscopy, and the purity was assessed by high performance liquid chromatography. We acquired Ag85A whole protein from Colorado State University through National Institutes of Health (NIH) Contract HHSN266200400091c, "TB vaccine testing and research materials." Isolation of MTB Ag85A from cell culture was performed as previously described [17]. Heat shock protein was provided as a kind gift from Doug Kernodle (Vanderbilt University). Due to limitations in cell numbers within the BAL, not all antigens were tested for each subject. The antigens were prioritized in the following order: ESAT-6, KatG, Ag85A, sodA and HSP.

**Table 2:** Peptide Sequence

Peptide		Sequence
Esat-6	14	NNALQNLARTISEAG
	15	NLARTISEAGQAMAS
KatG	31	WTNTPTKWDNSFLEI
	32	TKWDNSFLEILYGYE
Ag85A		Whole protein
	a	LQVPSPSMGRDIKVQFQSGG
	b	DWYQPACGKAGCQTYKWETL
	3	DIKVQFQSGGANSPALYLLD
	6	WDINTPAFEWYDQSGLSVVM
SodA	31	LGIVPLLLLDMWEHA
	33	MWEHAFYLQYKNVKV
	36	DFAKAFWNVVNWADV
	38	NWADVQSRYAAATSQ
HSP70		Whole protein

### Flow cytometry analysis - cytokine and proliferation assays

Intracellular staining was performed in order to identify IFN-γ secreting T cells in response to microbial stimulation. For the intracellular cytokine assay, 1-2×10^5 ^BAL cells were incubated in RPMI 1640 supplemented medium with or without antigen (ESAT-6, KatG, Ag85A, sodA or HSP peptides; 20 μg/ml) or staphylococcal enterotoxin B (SEB; 10 μg/ml; Sigma) as positive control and the anti-CD28 and anti-CD49d antibodies (1 μg/ml each; BD Biosciences) at 37°C under 5% CO_2 _for 1 hour before addition of BD GolgiStop (BD Biosciences). In addition, to assess for nonspecific recognition, neoantigen, Keyhole Limpet Hemocyanin (KLH; 10 μg/ml; Calbiochem) was included as an additional negative control. Following a 6 hour incubation at 37°C under 5% CO_2_, cells were washed and stained with the surface antibodies anti-CD3, anti-CD4, and anti-CD8 (BD Biosciences) at 4°C for 30 minutes. After washing, fixation, and permeabilization, using a Fix & Perm Kit according to the manufacturer's instructions (BD Biosciences), anti-IFN-γ and anti-IL-2 (BD Biosciences) were added at 4°C for 45 minutes. Cells were washed and analyzed via flow cytometry. The IFN-γ and IL-2 frequencies were defined as the subject's percentage of stimulated CD3+CD4+ or CD3+CD8+ T cells minus their unstimulated background frequency. Based upon previous studies, a response was considered positive when the frequency of recognition was at least twice background fluorescence and greater than 0.5% [18-20].

To determine proliferation and quantitate cell division, purified BAL cells were labeled with carboxyfluorescein succinimidyl ester (CFSE; Molecular Probes). Purified cells were first washed and resuspended in PBS. While vortexing the cells, CFSE was added at a final concentration of 5 μM. The mixture was vortexed for an additional 15 seconds and incubated at 37°C for 3 minutes. Labeling was quenched by the addition of 50% FBS in PBS. Cells were washed once more with 50% serum PBS, followed by two washes with RPMI 1640-supplemented medium. CFSE-labeled BAL cells were stimulated in RPMI 1640 supplemented medium with or without peptide (20 μg/ml) or SEB as positive control.

All flow cytometry experiments were acquired with a LSR-II flow cytometer (BD Biosciences). Live cells were gated based on forward and side scatter properties and analysis was performed using FlowJo software (Tree Star). A minimum of 30,000 events were acquired per sample.

### Statistical analysis

Statistical analyses were performed using statistical programming language R (version 2.8.1.). Association of a positive detection of immune responses to one or more mycobacterial virulence factors but not from other pulmonary disease controls (i.e. yes/no) was determined by using Fisher's exact test due to anticipated small cell counts. Differences in the distributions of immune recognition of mycobacterial antigen by CD4+ and CD8+ T cells from BAL were tested across diagnosis groups using the Wilcoxon rank sum test at an α-level of 0.05. In addition, it is unclear whether each radiographic stage of sarcoidosis reflects a different etiology or a distinct immune response to the same antigen. Therefore, differences in the distribution of immune recognition in sarcoidosis patients across two of four radiographic stages were assessed using the Kruskal-Wallis rank sum test. All tests of association were performed using an alpha-level of 0.05. No adjustments will be made for multiple comparisons.

## Results

### Sarcoidosis BAL CD4+ T cells exhibit multiple recognition of mycobacterial antigens

**Figure 1:**
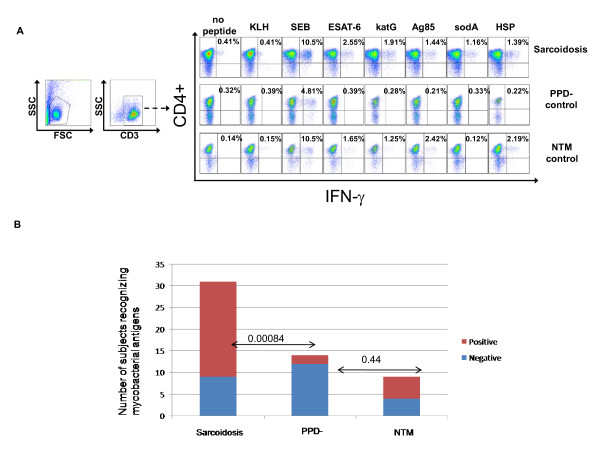
**Sarcoidosis CD4+ Th1 responses in BAL to multiple mycobacterial antigens**. BAL from sarcoidosis subjects, PPD- diseased controls and non-Tuberculosis Mycobacterial (NTM) infection subjects were stimulated with neoantigen (KLH), ESAT-6, katG, Ag85A, sodA, HSP and SEB (positive control), then intracellular cytokine staining for IFN-γ was performed. (A) Gating strategy and representative flow data for CD4+ T cell IFN-γ responses. (B) The number of Sarcoidosis, PPD- and NTM subjects that did (red) or did not (blue) respond to stimulation with mycobacteria peptides. CD4+ T cell response was defined as positive when the frequency of recognition was at least twice background fluorescence and greater than 0.5%.

Twenty-two of the 31 sarcoidosis BAL samples produced a CD4+ response to at least one of the mycobacterial epitopes of ESAT-6, KatG, Ag85A, sodA, or HSP, compared to two of 14 PPD- controls (p = 0.00084) and five of nine NTM controls (p = 0.437) (Figure 1A, B; Table 3). Nineteen of the 31 sarcoidosis patients responded to at least one of the two ESAT6 peptides compared with two of 14 PPD- controls (p = 0.0042) and five of nine NTM controls (p = 1.0). For the two katG peptides, 11 of 29 sarcoidosis subjects, two of 14 PPD- subjects (p = 0.16) and four of eight NTM subjects responded to at least one peptide (p = 0.68). Using four Ag85A peptides and Ag85A whole protein, 10 of 22 sarcoidosis subjects, one of 12 PPD- subjects (p = 0.053) and two of three NTM subjects responded to at least one of the peptides and/or whole protein (p = 0.59). Seven of 21 sarcoidosis subjects responded to at least one of four sodA peptides, compared with one of nine PPD- subjects (p = 0.37) and none of three NTM subjects (p = 0.53). Four of 17 sarcoidosis subjects responded to HSP, compared with one of seven PPD- subjects (p = 1) and one of three NTM subjects (p = 1).

**Table 3:** CD4+ and CD8+ T cell response to mycobacterial virulence factors

	Sarcoidosis	PPD-	NTM	P-value
Total Subjects	31	14	9	
CD4+ Response to any mycobacterial antigen	22	2	5	0.0008*
CD4+ Response to multiple mycobacterial antigens	16	2	4	0.02*
CD8+ Response to any mycobacterial antigen	18	2	3	0.009*
CD8+ Response to multiple mycobacterial antigen	12	2	3	0.12*

*Comparison of Sarcoidosis to PPD- subjects by two-tailed, Fisher's exact test. CD4+ or CD8+ response was defined as positive when the frequency of recognition was at least twice background fluorescence and greater than 0.5%.

**Figure 2:**
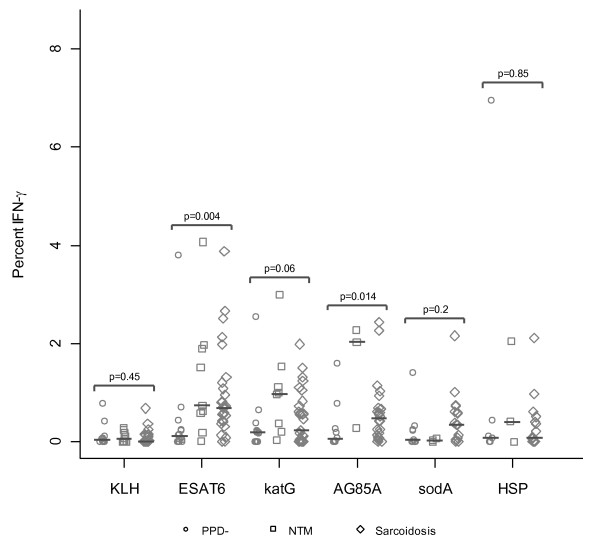
**Sarcoidosis CD4+ BAL T cells produce IFN-γ in response to multiple mycobacterial antigens**. Percent CD4+ cells that produce IFN-γ after stimulation with neoantigen (KLH), ESAT-6, katG, Ag85A, sodA, and HSP. Differences in the CD4+ immune response across diagnosis groups were noted using the Wilcoxon rank sum test. Medians are depicted by lines. Stimulation of BAL cells with SEB resulted in a positive IFN-γ response for sarcoidosis subjects and disease controls. The p values listed are for the comparison between sarcoidosis and PPD-. No significant difference was seen between sarcoidosis and NTM group.

When compared to PPD^- ^controls, there was a significant difference in the distribution of the sarcoidosis Th-1 immune response against ESAT-6 (p = 0.004, Wilcoxon), katG (p = 0.06, Wilcoxon), and Ag85A (p = 0.014, Wilcoxon) (Figure 2). Although sarcoidosis BAL-derived T cells produced more IFN-γ in response to sodA, there was not a significant difference from PPD- controls. Upon stimulation with mycobacterial HSP, sarcoid BAL-derived T cells did not produce more IFN-γ than PPD^- ^controls.

### Sarcoidosis BAL-derived CD8+ T cells recognize multiple mycobacterial antigens

**Figure 3:**
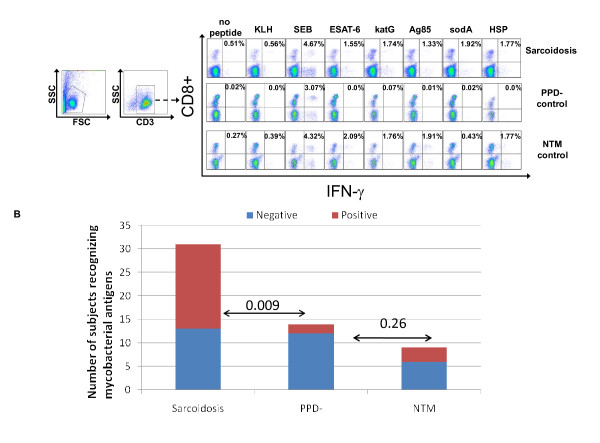
**Sarcoidosis CD8+ Th1 responses in BAL to multiple mycobacterial antigens**. BAL from sarcoidosis subjects, PPD- diseased controls and non-Tuberculosis Mycobacterial (NTM) infection subjects were stimulated with neoantigen (KLH), ESAT-6, katG, Ag85A, sodA, HSP and SEB (positive control), then intracellular cytokine staining for IFN-γ was performed. (A) Gating strategy and representative flow data for CD8+ T cell IFN-γ responses. (B) The number of Sarcoidosis, PPD- and NTM subjects that did (red) or did not (blue) respond to stimulation with mycobacteria peptides. CD8+ T cell response was defined as positive when the frequency of recognition was at least twice background fluorescence and greater than 0.5%.

Eighteen of the 31 sarcoidosis subjects produced a CD8+ response to at least one of the mycobacterial virulence factors: ESAT-6, KatG, Ag85A, sodA, or HSP, compared to two of 14 PPD- controls (p = .009) and three of nine NTM controls (Figure 3A,B; Additional File 1, Table S1) (p = 0.26). Seventeen of 31 sarcoidosis subjects responded to ESAT-6 peptides compared with two of 14 PPD- subjects (p = 0.021), and three of nine NTM subjects (p = 0.45). CD8+ T cells produced IFN-γ in response to katG in 8 of 29 sarcoidosis subjects, two of 14 PPD- subjects (p = 0.46) and three of eight NTM subjects (p = 0.67). Nine of 22 sarcoidosis subjects recognized at least of the four Ag85A peptides or whole protein, compared to two of 12 PPD- subjects (p = 0.14) and one of three NTM subjects (p = 1.0). Of the four sodA peptides, seven of 21 sarcoidosis subjects responded compared to one of nine PPD- subjects (p = 0.20), and none of three NTM subjects (p = 0.53). Using HSP, there was a response from 5 of 17 sarcoidosis patients, one of seven PPD- patients (p = 0.63) and one of three NTM patients. (p = 1.0). Of those who demonstrated CD8+ IFN-γ responses against mycobacterial virulence factors, 13 of 31sarcoidosis versus two of 14 PPD- controls (p = 0.09) and three of nine NTM controls (0.49) had responded to multiple antigens (Figure 3B).

**Figure 4:**
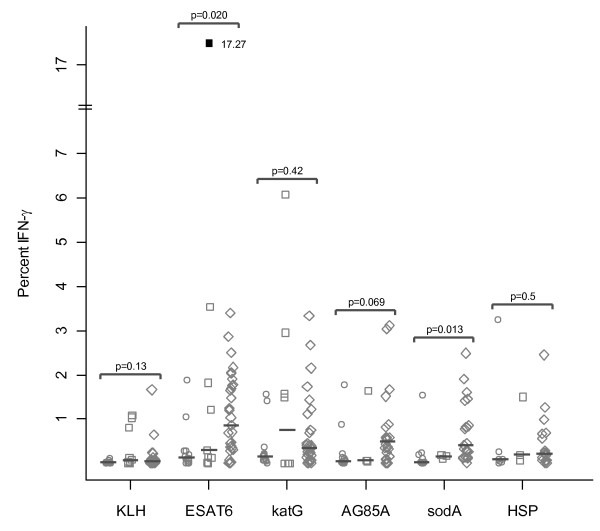
**Sarcoidosis CD8+ BAL T cells produce IFN-γ in response to multiple mycobacterial antigens**. Percent CD8+ cells that produce IFN-γ after stimulation with neoantigen (KLH), ESAT-6, katG, Ag85A, sodA, and HSP. Differences in the CD8+ immune response across diagnosis groups were noted using the Wilcoxon rank sum test. Medians are depicted by lines. Stimulation of BAL cells with SEB resulted in a positive IFN-γ response for sarcoidosis subjects and disease controls. The p values listed are for the comparison between sarcoidosis and PPD-. No significant difference was seen between sarcoidosis and NTM group.

In regards to the distribution of the CD8+ T cell response against mycobacterial virulence factors, IFN-γ immune responses against ESAT-6 (0.02, Wilcoxon) and sodA (0.013, Wilcoxon) were significant between sarcoidosis and PPD- subjects. The distribution of the immune responses against Ag85A approached significance among sarcoidosis and PPD- (0.069, Wilcoxon). There was no significant difference between these two groups in response to katG or HSP (Figure 4).

### Individual sarcoidosis subjects recognize multiple distinct mycobacterial epitopes

**Figure 5:**
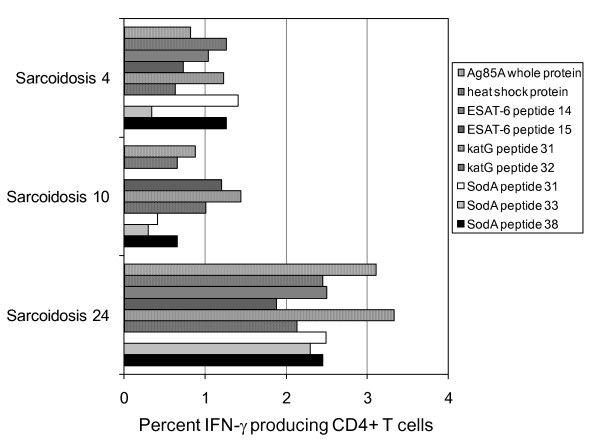
**Sarcoidosis CD4+ BAL T cells recognize multiple epitopes of mycobacterial proteins**. BAL T cells were stimulated with a panel of 9 mycobacterial peptides and intracellular cytokine staining was performed to measure the number of IFN-γ producing cells. Sarcoidosis 4, 10, and 24 CD4+, BAL-derived T cells were stimulated with all 9 mycobacterial peptides. Not only did these subjects recognize multiple mycobacterial peptides but also recognized multiple epitopes within the proteins. CD4+T cell response was defined as positive when the frequency of recognition was at least twice background fluorescence and greater than 0.5%.

The five mycobacterial virulence factors chosen for T cell stimulation demonstrated no similarity in peptide sequence (Table 2). Despite this lack of homology in position identity among the mycobacterial peptides, 16 sarcoidosis subjects, two PPD- controls and four NTM controls demonstrated CD4+ T cell responses to multiple antigens (Figure 5). Sarcoidosis 4 and Sarcoidosis 24 responded to all five antigens, demonstrating distinctions in the magnitude of the response to individual peptides (Figure 5). No single antigen was recognized by all sarcoidosis subjects. If one considers the 22 patients that demonstrated an immune responses to any mycobacterial antigen, 20 responded to ESAT-6; only two of 22 multiple responders did not respond to ESAT-6. Of the six subjects recognizing only one mycobacterial virulence factor, five responded to ESAT-6 and one to KatG. PPD- 11 was the lone subject with pneumonia who demonstrated strong responses against ESAT-6, KatG, Ag85A and HSP (data not shown).

Of those sarcoidosis subjects demonstrating CD8+ T cell responses to multiple antigens, Sarcoidosis 4, 8 and 24 responded to all five antigens, despite the fact that these peptides were not structurally similar (data not shown). No single antigen was recognized by every subject who demonstrated multiple responses. The majority of sarcoidosis subjects did respond to ESAT-6; only one of 13 multiple responders did not respond to ESAT-6. All five single responders demonstrated IFN-γ immune responses when stimulated by ESAT-6.

### Sarcoidosis BAL cells proliferate in response to mycobacterial antigen

**Figure 6:**
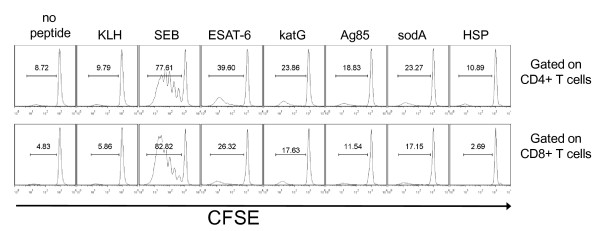
**Sarcoidosis BAL T cells proliferate in response to multiple mycobacterial antigens**. Sarcoidosis BAL cells were CFSE-labeled and activated with neoantigen (KLH), ESAT-6, katG, Ag85A, sodA, and HSP. Day 4 post-activation, antigen-specific proliferation of CD4+ and CD8+ T cells was assessed by gating on CD3+CD4+ T cells or CD3+CD8+ T cells and analyzing the CFSE expression of each subset by flow cytometry. Percent proliferation is indicated by bracket above peaks. Similar results were found in the three other tested subjects.

In chronic inflammatory states, non-specific recognition can induce cytokine production. Although the lack of recognition of KLH and the detection of immune responses to mycobacterial antigens suggested that the recognition observed was antigen-specific, we tested the ability of the BAL-derived T cells to proliferate in response to each of the five mycobacterial virulence factors. BAL cells were labeled with carboxyfluorescein succinimidyl ester (CFSE), a fluorescent dye used to monitor the number of cell divisions, then stimulated with the peptide or whole protein of interest on 5 sarcoidosis patients. In Sarcoidosis 8, activation with ESAT-6, katG, Ag85A, and sodA resulted in moderate proliferation of the CD4+ and CD8+ T cells compared to no peptide or KLH (neoantigen), while heat shock protein elicited low level proliferation (Figure 6). This lack of proliferation to heat shock protein was consistent with the lack of immune recognition observed in Sarcoidosis 8. These results, combined with the lack of recognition of KLH (Figure 6), provide compelling evidence of antigen-specific recognition of multiple mycobacterial antigens in diagnostic bronchoalveolar lavage, an active site of sarcoidosis involvement. Evidence of proliferation upon stimulation with mycobacterial antigens was also detected in Sarcoidosis 4, 10 and 24 (data not shown).

### CD4+ and CD8+ immune responses observed at radiographic stages of sarcoidosis

**Figure 7:**
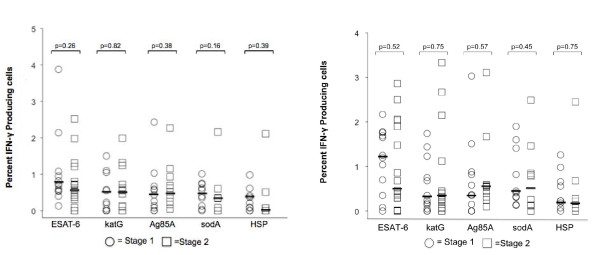
**Presence of mycobacterial responses does not correlate with radiographic stage**. Response to mycobacterial antigens was seen in radiographic stages 1-2. There were not enough subjects with stage 3-4 to assess differences in distribution of immune recognition across these stages. Kruskal-Wallis rank sum test was used to assess differences in distribution of immune responses. Medians depicted by lines. (A) CD4+ T cell response to ESAT-6, katG, Ag85A, sodA, and HSP. (B) CD8+ T cell response to ESAT-6, katG, Ag85A, sodA, and HSP.

We evaluated the immune responses to the multiple antigens in terms of Scadding radiograph stage I and II. There was no significant difference in the percentage or distribution of Th-1 cellular responses to mycobacterial antigen between these two stages (Figure 7). We did not include Scadding stage 0, III, and IV because of the paucity of subjects with these radiograph stages, compared to stage I and II. The analysis was performed on diagnostic BAL, and very few subjects presented with normal chest x-rays (Scadding stage 0) or advanced radiographic disease (Scadding stage III or IV).

## Discussion

The association of mycobacterial antigens with sarcoidosis pathogenesis continues to strengthen. Independent studies have demonstrated molecular and immunologic evidence of mycobacterial virulence factors in sarcoidosis pathogenesis [5,7]. This study is the first to report the presence of Th-1 immune responses to multiple mycobacterial virulence factors in sarcoidosis diagnostic bronchoalveolar lavage. This study also illustrates that rather than a single, persistent antigen serving as the target of the sarcoidosis adaptive immune response, this response is heterogeneous against multiple, distinct mycobacterial epitopes. This response is also distinct from PPD- subjects with alternative lung diseases such as Aspergillosis, fungal infection, and lymphoma but not from subjects with pulmonary infection due to non-tuberculous mycobacteria.

The observation of immune responses to these virulence factors is noteworthy for several reasons. It is possible that these immune responses reflect the presence of several persistent, poorly degradable, mycobacterial proteins after prior exposure to a pathogenic *Mycobacterium*. Previous studies in mice and humans have demonstrated the proteins such as ESAT-6, Antigen 85 and sodA are major T cell targets of the host immune response in humans and mice, eliciting a Th-1 immunophenotype [21]. A strong immune response against these virulence factors aid in protection against progressive infection. Prior reports of proteins such as ESAT-6, katG and sodA demonstrate that they are secreted through the mycobacterial secretion system. For example, in *M. tuberculosis*, the sodA protein is one of the few proteins identified as requiring SecA2 for its secretion [22]. It has been recently reported that it is the interaction of these secreted proteins, such as ESAT-6, with host epithelium that serve as the nidus for granuloma formation [23].

In this study, we report Th-1 responses against multiple, distinct mycobacterial antigens among sarcoidosis subjects (Figure 5). Of the mycobacterial peptides tested, ESAT-6 peptide was recognized most frequently by the sarcoidosis subjects (Figure 1). This increased recognition of ESAT-6 above other mycobacterial virulence factors has been previously reported in subjects with known mycobacterial infection [24,25]. One explanation for the increased recognition of ESAT-6 is that it contains epitopes that are recognized by a significant percentage of the most prevalent HLA types in the world [26]. It should be noted that the number of subjects that were tested using sodA and HSP was less than the number tested for other peptides due to limitations in BAL cells. It is possible that recognition of these peptides may have been significant if more subjects were tested. There were also distinctions in the magnitude of the response, as well as the number of subjects between the sarcoidosis and PPD- subjects (Figure 1 and 2). The magnitude of the response seen is consistent with what has been previously reported in subjects with active mycobacterial infection. A prior report noted the disappearance of sarcoidosis responses against katG upon clinical resolution [9], which suggests that they are important in sarcoidosis pathogenesis. We also noted disappearance of immune responses against katG and ESAT-6 in three sarcoidosis subjects as they resolved their disease (unpublished data). Immune responses against these mycobacterial antigens have also been reported in subjects with infection by known virulent mycobacteria [27,28]. The absence of immune responses to KLH, as well as the detection of proliferation upon stimulation by these antigens, also demonstrates that they are targets of the sarcoidosis adaptive immune response at the time of diagnosis (Figure 6).

## Conclusions

In summary, this investigation demonstrates that numerous epitopes of mycobacterial virulence factors are targets of the adaptive immune response, eliciting a Th-1 immunophenotype within CD4+ and CD8+ T cells. It is possible that other mycobacterial and human antigens not reported in this study are also targets of BAL-derived T cells. The magnitude of the response mimics those that have been reported in subjects with active mycobacterial infection, and warrants further investigation of the contribution mycobacteria have to sarcoidosis pathogenesis.

## Abbreviations used in this paper

BAL: bronchoalveolar lavage; NTM: non-tuberculosis mycobacteria; PPD: Purified Protein Derivative negative; ESAT-6: early secreted antigenic target protein 6; katG: catalase-peroxidase; Ag85A: mycolyl transferase Antigen 85A; sodA: superoxide dismutase A; HPS: heat shock protein; IFN-γ: interferon-γ; SEB: staphylococcal enterotoxin B; KLH: Keyhole Limpet Hemocyanin; CFSE: carboxyfluorescein succinimidyl ester.

## Competing interests

The authors declare that they have no competing interests.

## Authors' contributions

K A Oswald-Richter developed and coordinated the immunological experiments, performed data analysis, and contributed to the writing of the manuscript. D C Beachboard performed experiments, analyzed data, and contributed to the writing of the manuscript. X Zhan and C F Gaskill performed experiments. S Abraham processed and distributed BAL samples collected at Cleveland Clinic. C Jenkins performed statistical analysis for the study. D A Culver was responsible for study design and manuscript preparation. W Drake was the principal investigator and participated in the coordination of the study, data analysis, and the writing and editing of the manuscript. All authors read and approved the final manuscript.

## Supplementary Material

Additional file 1 **Complete Patient Data Table**. Patient data for all subjects included in study. Click here for file
