# Supplementary material for: Multiple mycobacterial antigens are targets of the adaptive immune response in pulmonary sarcoidosis
Source: Respir Res. 2010 Nov 23;11(1):161. doi: 10.1186/1465-9921-11-161 (PMC2999599; doi:10.1186/1465-9921-11-161)
Supplement: Additional file 1 — Complete Patient Data Table. Patient data for all subjects included in study. [file 1465-9921-11-161-S1.DOC]

| Subject  **Additional File 1, Table S1: Complete Patient Data DataSummary** | Age/Race/Sex | Diagnosis | HLA-DRB | IS at Bronch | Smoking Status | +CD4 Response to Mycobacterial Antigens | No. Responses/ No. tested | +CD8 Response to Mycobacterial Antigens | No. Responses/ No. tested | CD4/CD8 ratio |
| --- | --- | --- | --- | --- | --- | --- | --- | --- | --- | --- |
| Sarcoidosis 1 | 41CF | Sarcoidosis 0^1^ | 1501/1301 | No | EX^3^ | No | 0/5 | No | 0/5 | 1.20 |
| Sarcoidosis 2 | 52CF | Sarcoidosis 1 | ND^2^ | No | EX | Yes | 2/5 | Yes | 3/5 | 2.99 |
| Sarcoidosis 3 | 60CM | Sarcoidosis 1 | 0701/1302 | Yes | NS^4^ | Yes | 1/2 | Yes | 1/1 | 5.66 |
| Sarcoidosis 4 | 43CF | Sarcoidosis 1 | 04/1302 | Yes | NS | Yes | 5/5 | Yes | 5/5 | 40.90 |
| Sarcoidosis 5 | 33CM | Sarcoidosis 1 | 08/1401 | Yes | NS | Yes | 3/5 | Yes | 4/5 | 1.80 |
| Sarcoidosis 6 | 63AAM | Sarcoidosis 1 | 1201/1301 | Yes | EX | Yes | 2/5 | No | 0/5 | 2.21 |
| Sarcoidosis 7 | 32CF | Sarcoidosis 1 | 04/0101 | No | EX | Yes | 2/5 | Yes | 3/5 | 4.64 |
| Sarcoidosis 8 | 27CF | Sarcoidosis 1 | 1301/1501 | No | NS | Yes | 3/5 | Yes | 5/5 | 1.05 |
| Sarcoidosis 9 | 30AAM | Sarcoidosis 1 | 1302/1401 | Yes | EX | Yes | 2/3 | Yes | 2/3 | 2.24 |
| Sarcoidosis 10 | 27AAF | Sarcoidosis 1 | 1101/1303 | No | NS | Yes | 4/5 | Yes | 5/5 | 15.83 |
| Sarcoidosis 11 | 47CF | Sarcoidosis 1 | ND | No | NS | Yes | 2/4 | Yes | 3/4 | 1.06 |
| Sarcoidosis 12 | 37CF | Sarcoidosis 1 | 04/1301 | No | AS^5^ | No | 0/5 | No | 0/5 | 4.25 |
| Sarcoidosis 13 | 48CF | Sarcoidosis 1 | 1101 | Yes | NS | No | 0/5 | No | 0/5 | 6.61 |
| Sarcoidosis 14 | 42AAF | Sarcoidosis 2 | ND | No | NS | No | 0/5 | Yes | 1/5 | 0.64 |
| Sarcoidosis 15 | 61AAM | Sarcoidosis 2 | 0301/0101 | No | EX | Yes | 2/3 | No | 0/3 | 3.76 |
| Sarcoidosis 16 | 28CF | Sarcoidosis 2 | 0301/1301 | No | AS | No | 0/2 | No | 0/2 | 3.2 |
| Sarcoidosis 17 | 40CF | Sarcoidosis 2 | 1301 | Yes | EX | No | 0/2 | No | 0/2 | 2.6 |
| Sarcoidosis 18 | 36AAF | Sarcoidosis 2 | 08/0701 | No | AS | Yes | 1/2 | Yes | 2/2 | 2.5 |
| Sarcoidosis 19 | 47CM | Sarcoidosis 2 | ND | No | NS | No | 0/2 | No | 0/2 | 5.1 |
| Sarcoidosis 20 | 49CM | Sarcoidosis 2 | ND | Yes | EX | No | 0/4 | No | 0/4 | 2.8 |
| Sarcoidosis 21 | 27H/AAF | Sarcoidosis 2 | 04/1401 | No | EX | Yes | 3/5 | Yes | 4/5 | 9.36 |
| Sarcoidosis 22 | 52AAF | Sarcoidosis 2 | 0302/12 | Yes | NS | Yes | 1/5 | No | 0/5 | 1.17 |
| Sarcoidosis 23 | 50CF | Sarcoidosis 2 | 1101/1502 | Yes | NS | Yes | 2/5 | Yes | 2/5 | 0.75 |
| Sarcoidosis 24 | 53AAF | Sarcoidosis 2 | ND | Yes | EX | Yes | 5/5 | Yes | 5/5 | 0.83 |
| Sarcoidosis 25 | 54CM | Sarcoidosis 2 | ND | Yes | NS | Yes | 1/4 | No | 0/4 | 0.56 |
| Sarcoidosis 26 | 53CM | Sarcoidosis 2 | ND | No | EX | Yes | 2/4 | Yes | 2/4 | 5.10 |
| Sarcoidosis 27 | 43CF | Sarcoidosis 2 | 0701/1101 | No | NS | No | 0/3 | No | 0/3 | 2.87 |
| Sarcoidosis 28 | 60CM | Sarcoidosis 2 | ND | No | NS | Yes | 2/5 | Yes | 2/5 | 70.54 |
| Sarcoidosis 29 | 25CF | Sarcoidosis 2 | ND | No | NS | Yes | 1/1 | Yes | 1/1 | 1.35 |
| Sarcoidosis 30 | 54CM | Sarcoidosis 2 | ND | No | NS | Yes | 1/2 | No | 0/2 | 7.21 |
| Sarcoidosis 31 | 64CM | Sarcoidosis 3 | 0401/1501 | No | NS | Yes | 1/2 | Yes | 1/2 | 4.1 |
| PPD- 1 | 52AAM | Idiopathic cardiomyopathy | ND |  | AS | No | 0/5 | No | 0/5 | 1.5 |
| PPD- 2 | 32CF | Asthma | ND |  | EX | No | 0/3 | No | 0/3 | 1.62 |
| PPD- 3 | 57CF | Fungal infection | ND |  | EX | No | 0/5 | No | 0/5 | ND |
| PPD- 4 | 24CF | Lymphoma | ND |  | NS | No | 0/5 | No | 0/5 | 0.15 |
| PPD- 5 | 49A/SF | Adenocarcinoma | ND |  | EX | Yes | 4/5 | Yes | 4/5 | 0.55 |
| PPD- 6 | 49CM | Aspergillus infection | ND |  | EX | No | 0/3 | No | 0/3 | 0.19 |
| PPD- 7 | 61CF | Interstitial Lung disease | ND |  | NS | No | 0/1 | No | 0/1 | 2.89 |
| PPD- 8 | 42CF | Lung transplant | ND |  | NS | No | 0/3 | No | 0/3 | 0.54 |
| PPD- 9 | 59CF | Lung transplant | ND |  | NS | No | 0/5 | No | 0/5 | 0.15 |
| PPD- 10 | 47AAM | Hemotysis | ND |  | NS | No | 0/5 | No | 0/5 | 0.14 |
| PPD- 11 | 56AAF | Pneumonia | ND |  | NS | Yes | 4/5 | Yes | 4/5 | 36.67 |
| PPD- 12 | 29CF | Granulomatus infamation | ND |  | NS | No | 0/4 | No | 0/4 | 1.7 |
| PPD- 13 | 50CF | Anti-synthetase syndrome | ND |  | EX | No | 0/2 | No | 0/2 | 1.4 |
| PPD- 14 | 25CF | Diagnosis not obtained | ND |  | EX | No | 0/4 | No | 0/4 | 1.3 |
| NTM 1 | 67CF | Chronic hypersensativity pneumonitis | ND |  | EX | No | 0/2 | No | 0/2 | 3.3 |
| NTM 2 | 72CF | Chronic hypersensativity pneumonitis | ND |  | EX | No | 0/2 | No | 0/2 | 9.1 |
| NTM 3 | 58CF | Chronic hypersensativity pneumonitis | ND |  | EX | Yes | 2/5 | Yes | 2/5 | ND |
| NTM 4 | 75CF | Non-tuberculosis mycobacterium (no speciation) | ND |  | EX | No | 0/2 | No | 0/2 | 2.3 |
| NTM 5 | 65CM | *M. avium* infection | ND |  | EX | Yes | 2/2 | Yes | 2/2 | 4.2 |
| NTM 6 | 70CM | *M. avium* infection | ND |  | NS | No | 0/2 | No | 0/2 | 2.6 |
| NTM 7 | 51CF | M. mucogenicum | ND |  | NS | Yes | 4/5 | Yes | 4/5 | 9.01 |
| NTM 8 | 65CM | HIV+/inactive TB | ND |  | AS | Yes | 3/5 | No | 0/5 | 0.49 |
| NTM 9 | 71AAM | Lung nodule | ND |  | NS | Yes | 1/1 | No | 0/1 | 1.18 |

^1^ Scadding Radiographic stage. ^2^Not determined. ^3^EX = ex-smoker; ^4^NS = never smoker. ^5^AS = active smoker;
